# Exploring the ‘fit for work’ principle: The association between occupational physical activity, cardio-respiratory fitness, and mortality – a meta-analysis of male worker data

**DOI:** 10.5271/sjweh.4218

**Published:** 2025-05-01

**Authors:** Margo Ketels, Bart Cillekens, Els Clays, Maaike A Huysmans, Dirk De Bacquer, Andreas Holtermann, Richard P Troiano, Paul Jarle Mork, Steinar Krokstad, Henry Völzke, Marcus Dörr, Martin Bahls, Till Ittermann, Johan Clausen, Magnus T Jensen, Jussi Kauhanen, Ari Voutilainen, Miriam Wanner, Matthias Bopp, Willem van Mechelen, Allard J van der Beek, Pieter Coenen

**Affiliations:** 1 Department of Public Health and Primary Care, Ghent University, Ghent, Belgium.; 2 Amsterdam UMC, Vrije Universiteit Amsterdam, Department of Public and Occupational Health, Amsterdam Public Health Research Institute, Amsterdam, The Netherlands.; 3 National Research Centre for the Working Environment, Copenhagen, Denmark.; 4 Department of Sports Science and Clinical Biomechanics, University of Southern Denmark, Odense, Denmark.; 5 U.S. Public Health Service (retired), Arlington, VA, USA.; 6 Department of Public Health and Nursing, Norwegian University of Science and Technology, Trondheim, Norway.; 7 Levanger Hospital, Nord-Trøndelag Hospital Trust, Levanger, Norway.; 8 Department of Internal Medicine B (Cardiology), University Medicine Greifswald, Greifswald, Germany.; 9 German Centre for Cardiovascular Research (DZHK), partner site Greifswald, Greifswald, Germany.; 10 Institute for Community Medicine; University Medicine Greifswald, Greifswald, Germany.; 11 Epidemiological Research Unit, Departments of Occupational and Environmental Medicine, Bispebjerg University Hospital, Copenhagen, Denmark.; 12 Steno Diabetes Center Copenhagen, Herlev, Denmark.; 13 The Copenhagen City Heart Study, Copenhagen, Denmark.; 14 Institute of Public Health and Clinical Nutrition, University of Eastern Finland, Kuopio, Finland.; 15 University of Zürich, Epidemiology, Biostatistics and Prevention Institute, Zürich, Switzerland.; 16 University Hospital Zürich, Cancer Registry Zurich, Zug, Schaffhausen & Schwyz, Institute of Pathology & Molecular Pathology, Zürich, Switzerland.

**Keywords:** individual participant data, Human Activities, Death Rate, Physical Fitness, Population Characteristics, Ergonomics

## Abstract

**Objectives:**

This individual participant data meta-analysis investigates the association between occupational physical activity (OPA) and both cardiovascular mortality and all-cause mortality across different cardio-respiratory fitness (CRF) groups among male workers.

**Methods:**

Data were pooled from five European cohort studies. OPA was categorized into three levels and CRF into low, moderate, and high tertiles. OPA was assessed using self-reports and CRF through objective measurements. Two-stage meta-analyses were conducted. First, we analyzed each cohort using Cox-regression models then we pooled results with random effects model to evaluate the associations between OPA and both cardiovascular and all-cause mortality, stratified by CRF. Models were adjusted for age, body mass index, smoking status, leisure-time physical activity, and educational level.

**Results:**

Among 9922 men (mean age 46.8, standard deviation 6.7, years), 55.7% died during an average 25.6-year follow-up, of which 29.3% died from cardiovascular causes. Individuals with low CRF and high levels of OPA showed increased risks of cardiovascular [hazard ratio (HR) 1.27, 95% confidence interval (CI) 1.04–1.55] and all-cause mortality (HR 1.22, 95% CI 1.07–1.40) compared to those with low CRF and low levels of OPA. High CRF mitigated cardiovascular mortality risk (HR 1.08, 95% CI 0.79–1.48) but not all-cause mortality (HR 1.27, 95% CI 0.98–1.83) for those with high OPA.

**Conclusions:**

Our findings for cardiovascular mortality suggest that high CRF levels may protect workers with physically demanding jobs from adverse cardiovascular outcomes, supporting the ‘fit for work’ principle. However, this protective effect was not observed for all-cause mortality.

In the coming decades, the (Western) workforce will be confronted with numerous challenges. An aging population, a rising retirement age, and an increase in chronic, lifestyle-related diseases at a younger age (1, 2) will strain resources. Additionally, there is a shortage of workers in crucial sectors such as care services, manufacturing, and service industries (3). Workers in these fields typically come from lower socioeconomic backgrounds (4) and are exposed to high levels of occupational physical activity (OPA), potentially involving various health challenges (5–8).

Although leisure-time physical activity (LTPA) has widely been acknowledged as an important determinant of good health and longer life expectancy (9, 10), several studies have demonstrated that the health effects of physical activity could differ by domain. The health effects of OPA might be different from those of LTPA (6, 11). This phenomenon is referred to in the scientific literature as the ‘physical activity health paradox’ (12–15). Individuals regularly involved in high levels of OPA may not experience the same health-promoting effects as derived from LTPA (15) and might even face elevated risks of cardiovascular diseases and all-cause mortality (14, 16). A study involving over 500 000 workers in Sweden highlighted that those in physically demanding jobs often exhibited lower cardiorespiratory fitness (CRF) expressed by directly measured VO_2_max (Åstrand submaximal cycle ergometer test) and had worse health prognoses compared to white-collar workers (10, 17, 18). Possible explanations for the adverse health effects associated with OPA may be that it consists mainly of anaerobic physical activity combined with heavy lifting and/or repetitive movements (19). The intensity of these types of activities is often not high enough to induce a beneficial effect on CRF. Moreover, OPA typically takes place over extended periods (eg, 8 hours per working day) with insufficient recovery breaks. This may lead to increased blood pressure and heart rate, which in turn might result in an increased risk of developing cardiovascular disease (20).

Given the aforementioned findings and the considerable number of workers exposed to high levels of OPA, it is worthwhile to further investigate the potential counterintuitive association between OPA, cardiovascular mortality, and all-cause mortality. One way to enhance the body’s resilience against the negative impact of high OPA might be to enhance CRF. In fact, robust evidence demonstrates that higher levels of CRF are associated with reduced cardiovascular risk markers, morbidity and mortality (21–23). By contrast, low levels of CRF due to a lack of regular physical activity or exercise have been associated with the progression of atherosclerosis (20), higher risk of coronary heart disease, cardiovascular disease events and all-cause mortality (22). Thus, improving CRF by performing regular physical activity and/or exercise might be a promising intervention strategy to counteract the potentially negative effects of OPA on several health-related outcomes. Previous research has provided valuable insights into the moderating role of CRF in the association between OPA and mortality. Holtermann et al (21, 24) have demonstrated an increased risk of the combination of high OPA and low CRF on ischemic heart disease mortality and cardiovascular mortality. Similarly, albeit to a lesser extent, associations were found with all-cause mortality. Clays et al (25) found that among men with low CRF, those with high OPA and low LTPA had the highest all-cause mortality rates.

The ‘fit for work’ paradigm emphasizes that individuals who have good physical fitness levels, particularly cardiorespiratory fitness, are better equipped to handle the physical demands of their work than those with poor physical fitness levels (26). This paradigm suggests that being physically fit can help to mitigate the health risks associated with physically demanding work, potentially reducing the likelihood of injuries and premature death.

Leveraging data from the extensive *Active Worker Consortium*, a collaborative effort involving multiple research institutions and databases focused on workers from various countries, our study provides an opportunity to delve deeper into the associations between OPA, CRF, and mortality. Our objective is to explore the association between OPA and both cardiovascular and all-cause mortality within different CRF groups. Building upon the already existing body of evidence (24, 25), we hypothesize that individuals with high levels of OPA and low levels of CRF have an increased risk of cardiovascular and all-cause mortality compared to those with high levels of both OPA and CRF.

## Methods

### The Active Worker Consortium

The protocol for this study, which relies on data from the *Active Worker Consortium*, has been registered in PROSPERO (27). Further details about the study protocol of the *Active Worker Consortium* have been described in a protocol paper (27). The study was conducted in accordance with methods described by the Cochrane Individual Participant Data Meta-analysis Methods Group and PRISMA-IPD statement (28). The Medical Ethical Committee of Amsterdam UMC declared that the Medical Research Involving Human Subjects Act does not apply to this study (reference no. 2018.068). Data stem from previously conducted eligible cohort studies, both published and unpublished. The systematic search for original prospective cohort studies with data on OPA, LTPA, socioeconomic status (as determined by income, education level and/or social class), and all-cause and/or cardiovascular mortality in adult workers (18–65 years of age at the time of physical activity assessment) has been described in more detail before (29, 30) and has resulted in the selection of 49 unique cohort studies (from 106 articles).

### Data

Data were retrieved from the cohort studies [a full list can be found in the papers of Coenen et al (27, 29)] and were harmonized by labeling and recoding all requested variables. For the present study, an additional selection criterion was the availability of objectively measured CRF data. Based on this selection criterion, only five studies qualified for inclusion. This resulted in a subset of studies from the *Active Worker Consortium,* namely the Belgian Physical Fitness Study (BELFIT) (6), the Kuopio Ischemic Heart Disease Risk Factor (KIHD) study in Finland (31), the Study of Health in Pomerania (SHIP-START1) in Germany (32), the National Research Program (NRP) 1A study in Switzerland (33), and the Copenhagen male study in Denmark (34). Among these studies, three were initiated in the 1970s, one in the 1980s, and one in the 2000s (see supplementary material, www.sjweh.fi/article/4218, table S1 for more details). Most studies of this sample either lacked data on female participants or had insufficient data points to estimate hazard ratios (HR) for females. Consequently, we analyzed data from male participants only. Exclusion of the participants with no follow-up information or missing data for OPA, CRF, and cause of death and/or all-cause mortality resulted in a final dataset with data from 9922 male participants.

### Occupational physical activity (OPA)

In the original studies, OPA was defined and measured in various ways, including both categorical and continuous variables. Contrary to the approach proposed in the protocol paper (27), and our publications on the IPD (29, 30), this meta-analysis divided OPA into three rather than four categories as two of the five studies used only three OPA categories: (i) low physical activity (eg, work that involves mainly sitting, standing, or walking without lifting or carrying loads), (ii) moderate physical activity (eg, work that involves carrying light objects or climbing stairs), and (iii) high-level physical activity (eg, physically demanding tasks with frequent carrying or lifting of heavy loads). In cases where studies relied on four OPA categories, we merged the sedentary and low OPA groups into a single “low activity” category.

### Cardiovascular and all-cause mortality

The data on cardiovascular and all-cause mortality were registry- or hospital-based, and harmonized as a dichotomous variable depicting the incidence of the event (yes/no) and as a continuous variable depicting the time to event (expressed in days). More detailed information on the outcomes assessment and the International Classification of Diseases (ICD) codes used for cardiovascular mortality is shown in supplementary table S1. The BELFIT, KIHD, NRP 1A, and the Copenhagen Male studies, covered all cardiovascular diseases (ICD-10: I00–I99). In contrast, the SHIP-START1 study targeted specific subsets, such as hypertension (ICD-10: I10) and ischemic heart diseases (ICD-10: I20–I25).

### Cardiorespiratory fitness (CRF)

In the original studies, CRF was objectively assessed using various methods. Additional details about these methods can be found in supplementary table S3. To harmonize the data across original studies, participants were categorized into three equal CRF groups based on tertiles within each original study: low, medium, and high.

### Confounding variables

In all original cohort studies, age was measured as a continuous variable (in years). Marital status was harmonized as a dichotomous variable with the options ‘married/living together with a partner’ and ‘single’ (including no relationship, divorced or widow/widower). Educational level was harmonized into three levels: low (pre-primary, primary, and lower secondary), moderate (upper secondary), and high (post-secondary) education. Body mass index (BMI) was assessed in kg/m^2^; we excluded values <14 and >48 kg/m^2^. Blood pressure (systolic and diastolic) was harmonized as a continuous variable (in mmHg). Smoking was dichotomized into groups of current smokers and current non-smokers, the latter also including those who had smoked in the past. For LTPA, we harmonized data into four categories: (i) almost no regular physical activity, spending most leisure-time sitting (sedentary level), (ii) occasionally engaging in leisure-time activities such as slow walking or household activities (low level), (iii) engaging in activities such as brisk walking or dancing (moderate level), and (iv) regular engagement in activities such as jogging or cycling (high level).

### Risk of bias assessment

In order to address the risk of bias, two reviewers independently examined each of the studies using a published Cochrane scoring system (35). The criteria related to study participation, attrition, predictor variable measurements (scoring the assessment of both OPA and LTPA, with the score depending on the weakest assessment method) and outcomes. Any conflicts were resolved during a consensus meeting. More details can be found in supplementary table S2.

### Data analysis

Participant characteristics were presented by proportions and percentages for categorical variables and by means and standard deviations for continuous variables. We performed a two-stage meta-analysis to assess the association between OPA and both cardiovascular and all-cause mortality, stratified across the three CRF groups (ie, low, medium, and high). In the first stage, we analyzed each study separately. For four studies, we had individual participant data (IPD) (total N=4712). The participating researchers of this study analyzed one study remotely (N=5210). In the second stage of our analyses, results were pooled using the Stata *admetan* function. Random effects models were used due to high statistical heterogeneity (I^2^≥70%) (29). Multivariable survival Cox regression analyses were used to estimate HR with 95% confidence intervals (CI) with low OPA as the reference category. Adjustment for confounders was done stepwise. The first models were unadjusted, the second models were adjusted for BMI, age, smoking and LTPA and the third model was additionally adjusted for level of education. P<0.05 was considered to be statistically significant. All analyses were conducted with Stata, version 17 (StataCorp, College Station, TX, USA).

## Results

### Baseline characteristics

Table 1 provides an overview of the baseline characteristics of the 9922 male participants across the five distinct studies as well as for the entire IPD dataset. These characteristics encompass socio-demographic factors, cardiovascular risk factors, mortality rates, and physical activity level in both work and leisure. In our sample of male employees, the mean age at baseline was 46.8 (SD 6.7) years; 55.7% of the men died from all-causes of which 29.3% died from cardiovascular mortality over an average follow-up period of 25.6 (SD 8.2) years.

**Table 1:** Baseline socio-demographic, cardiovascular risk factors, mortality rates, and physical activity characteristics of the five studies separately and for the total dataset (N=9922). [SD=standard deviation; DNR=the cohort did not provide us with this information; NA=not available].^a^

Characteristics		BELFIT ^b^ (N=1498)			Copenhagen male ^c^(N=5210)			KIHD ^d^ (N=1737)			SHIP-START1 ^e^ (N=437)			NRP 1A ^f^ (N=1040)			Total (N=9922)	
		Mean (SD)	%		Mean (SD)	%		Mean (SD)	%		Mean (SD)	%		Mean (SD)	%		Mean (SD)	%
Age (years)		46.3 (4.2)			48.8 (5.3)			52.0 (4.9)			44.8 (9.4)			40.5 (10.9)			47.9 (5.9)	
Body mass index (BMI) (kg/m^2^)		25.5 (2.9)			25.3 (3.0)			26.8 (3.4)			27.6 (3.7)			24.7 (3.0)			25.6 (3.1)	
Follow-up duration (years)		16.9 (3.3)			28.4 (6.5)			27.8 (5.4)			8.2 (1.4)			32.5 (7.8)			25.5 (5.9)	
Systolic blood pressure (mmHg)		131.9 (13.4)			135.1 (DNR)			133.3(15.4)			132.5 (14.4)			128.1 (13.1)			131.6 (14.1)	
Diastolic blood pressure (mmHg)		82.1 (10.5)			83.2 (DNR)			88.8 (9.9)			84.6 (9.1)			80.4 (9.6)			84.4 (9.9)	
Cardiorespiratory fitness ^g^																		
	Overall	112.0 (21.0)			DNR			31.6 (7.1)			30.4 (6.9)			37.6 (9.3)			NA	
	1^st^ tertile	88.8 (8.9)			DNR			23.7 (3.7)			23.3 (2.7)			28.7 (4.1)			NA	
	2^nd^ tertile	112.0 (6.7)			DNR			31.3 (1.7)			29.7 (1.8)			36.8 (1.9)			NA	
	3^rd^ tertile	135.0 (13.5)			DNR			38.6 (4.7)			38.3 (4.6)			7.9 (6.9)			NA	
Smoking (yes)			45.6			72.0			30.5			30.4			49.9			56.6
Education level																		
	Low		25.7			55.2			53.5			14.6			12.7			44.4
	Moderate		62.5			28.1			39.9			58.1			55.4			39.4
	High		11.8			16.7			6.7			27.2			31.9			16.2
Marital status																		
	Single		7.3			6.0			11.7			19.2			NA			8.0
	Married or living together		92.7			94.0			88.3			80.8			NA			92.0
Leisure-time physical activity (LTPA)																		
	Sedentary		23.0			NA			27.2			23.1			NA			9.4
	Low		23.7			18.0			26.3			24.5			19.6			20.8
	Moderate		26.8			73.0			25.2			26.3			63.5			54.3
	High		26.5			9.0			21.4			26.1			16.9			15.5
Occupational physical activity (OPA)																		
	Low		75.6			29.0			50.3			44.0			34.2			41.1
	Moderate		12.5			51.0			25.0			26.8			48.9			39.2
	High		11.9			17.0			24.7			29.1			16.9			18.1
All-cause mortality																		
	Yes		10.2			92.1			49.7			2.5			40.7			63.3
	No		89.8			7.9			50.4			97.5			59,3			36.7
Cardiovascular mortality																		
	Yes		2.6			42.6			21.9			0.5			14.1			28.4
	No		97.4			57.4			78.1			99.5			85.9			71.6

^a^ The number of participants varies across variables due to missing data. The percentages of missing data are as follows: age=0.02%, BMI=0.15%, smoking=0%, education=0.77%, marital status=12.1%. All studies provided data only for participants with complete information on at least OPA, LTPA, and mortality. However, the Copenhagen male study also reported descriptive data for a small proportion of participants with missing values for LTPA (1.43%) and OPA (1.29%). ^b^ The Belgian Physical Fitness Study, Clays et al (6). ^c^ The Copenhagen male study, Holtermann et al (34). ^d^ The Kuopio Ischemic Heart Disease Risk Factor Study, Krause et al (31). ^e^ The Study of Health in Pomerania, Bahls et al (32). ^f^ National Research Program, Wanner et al (33). ^g^ For the BELFIT study, CRF was measured as watts/kg, all other studies for VO^2^max (ml.kg-1. min.1) (see supplementary table S3).

Table 2 shows lifestyle and other characteristics of males belonging to groups with different levels of CRF (based on tertiles). Based on these descriptive statistics, it can be observed that the group with the highest CRF generally smoked less, was slightly younger and higher educated, and had lower blood pressure compared to the other two groups.

**Table 2:** Baseline socio-demographic, cardiovascular risk factors, mortality rates, and physical activity characteristics according to cardiorespiratory fitness level. [SD=standard deviation; BMI=body mass index; OPA=occupational physical activity; LTPA=leisure-time physical activity; Syst BP=systolic blood pressure; Dia BP=diastolic blood pressure; CVD=cardiovascular disease; CRF=cardiorespiratory fitness].^a^

Characteristics		Level of cardiorespiratory fitness							
		CRF 1^st^ tertile (N=1575, 33.4%)			CRF 2^nd^ tertile (N=1589, 33.7%)			CRF 3^rd^ Tertile (N=1548, 32.9%)	
		Mean (SD)	% ^b^		Mean (SD)	% ^b^		Mean (SD)	% ^b^
Age (years)		48.8 (7.8)			46.9 (8.1)			45.17 (8.5)	
BMI (kg/m^2^)		26.7 (3.8)			25.9 (3.0)			25.27 (2.9)	
Syst BP (mm/hg)		134.1 (15.0)			131.7 (14.0)			129.1 (13.4)	
Dia BP (mm/hg)		85.7 (10.9)			84.1 (10.4)			83.3 (10.2)	
Smoking									
	Yes		43.4			42.0			33.2
	No		56.6			58.0			66.8
Education									
	Low		37.2			33.0			29.8
	Medium		32.4			34.8			32.8
	High		29.3			31.0			39.7
Marital Status									
	Single		32.1			28.7			30.7
	Married/living together		67.9			71.3			69.3
LTPA									
	Sedentary		25.1			19.3			14.0
	Low		26.2			24.8			20.3
	Moderate		32.2			34.6			36.0
	High		16.5			21.3			29.7
OPA									
	Low		55.9			55.5			54.5
	Moderate		26.9			25.5			25.5
	High		17.3			19.0			20.1
Mortality									
	Yes		41.4			29.8			21.0
	No		58.6			70.2			79.0
CVD Mortality									
	Yes		18.2			10.3			7.6
	No		81.8			89.9			92.4

^a^ These analyses are based only on four studies in our individual participant data. We did not have these characteristics for the Copenhagen Male study. b Frequency.

### Cardiovascular and all-cause mortality

Table 3 reports both crude and adjusted associations of OPA and the risk of cardiovascular mortality, stratified by CRF level. In the stratum of the low CRF group, the adjusted model (ie, model 3) showed that those with high OPA had a significantly increased risk of cardiovascular mortality compared to those with low OPA levels (HR 1.27, 95% CI 1.04–1.55). In the moderate and high CRF group, those with high OPA levels did not have a significantly increased risk of cardiovascular mortality compared to those with low OPA levels (HR 1.15, 95% CI 0.93–1.43 and HR 1.08, 95% CI 0.79–1.48, respectively). Forest plots of model 3 cardiovascular mortality are shown in supplementary figure S2.

**Table 3:** Occupational physical activity (OPA) and risk of cardiovascular mortality according to cardiorespiratory fitness (CRF) level: lowest, medium and highest tertile. [N=number of studies; n=number of participants ;CI=confidence interval; HR=hazard ratio]. **Bold denotes P<0.05.**

OPA		Cardiovascular mortality											
		N	n	HR ^a^	95% CI	N	n	HR ^b^	95% CI	N	n	HR ^c^	95% CI
Low CRF group													
	Low	4	1371	1		4	1368	1		4	1358	1	
	Moderate	4	1269	**1.27**	**1.11–1.45**	4	1266	**1.22**	**1.06–1.40**	4	1254	1.14	0.98–1.32
	High	4	493	**1.41**	**1.18–1.67**	4	492	**1.42**	**1.20–1.69**	4	448	**1.27**	**1.04–1.55**
Moderate CRF													
	Low	4	1380	1		4	1314	1		4	1300	1	
	Moderate	4	1322	**1.30**	**1.12–1.50**	4	1282	**1.30**	**1.11–1.53**	4	1270	1.14	0.78–1.68
	High	3	502	**1.49**	**1.23–1.81**	3	497	**1.44**	**1.18–1.75**	3	497	1.15	0.93–1.43
High CRF													
	Low	4	1229	1		4	1229	1		4	1221	1	
	Moderate	4	1208	1.13	0.95–1.34	4	1207	1.13	0.95–1.33	4	1198	1.08	0.90–1.30
	High	4	615	1.23	0.93–1.62	4	614	1.19	1.00–1.43	4	607	1.08	0.79–1.48

^a^ Model 1 = unadjusted. ^b^ Model 2 = adjusted for age, body mass index, smoking and leisure-time physical activity. ^c^ Model 3 = model 2 + additionally adjusted for educational level.

Table 4 reports results for all-cause mortality and shows a less clear pattern in the association between OPA and all-cause mortality across the different CRF strata compared to cardiovascular mortality. The findings for all-cause mortality indicate an increased risk of all-cause mortality with higher OPA levels across all CRF strata, but this risk was only statistically significant in the low CRF group (low CRF–high OPA: HR 1.22, 95% CI 1.07–1.40; moderate CRF–high OPA: HR 1.16, 95% CI 0.97–1.39; and high CRF–high OPA: HR 1.27, 95% CI 0.98–1.83). Forest plots of model 3 all-cause mortality are shown in supplementary figure S3.

**Table 4:** Occupational physical activity (OPA) and risk of all-cause mortality according to cardiorespiratory fitness (CRF) level: lowest, medium, and highest tertile. [CI=confidence interval; HR=hazard ratio; N=number of studies included; n=number of participants]. Bold denotes P<0.05.

OPA		All-cause mortality											
		N	n	HR ^a^	95% CI	N	n	HR ^b^	95% CI	N	n	HR ^c^	95% CI
Low CRF													
	Low	5	1437	1		5	1432	1		5	1422	1	
	Moderate	5	1340	**1.23**	**1.12–1.34**	5	1306	**1.21**	**1.09–1.33**	5	1294	**1.12**	**1.01–1.25**
	High	5	576	**1.31**	**1.16–1.48**	5	532	**1.36**	**1.20–1.54**	5	528	**1.22**	**1.07–1.40**
Moderate CRF													
	Low	5	1380	1		5	1379	1		5	1369	1	
	Moderate	4	1287	1.12	0.95–1.32	4	1282	**1.19**	**1.07–1.31**	4	1270	1.14	0.91–1.43
	High	5	606	**1.40**	**1.24–1.59**	5	605	**1.35**	**1.01–1.81**	5	599	1.16	0.97–1.39
High CRF													
	Low	5	1229	1		5	1228	1		5	1221	1	
	Moderate	4	1208	1.04	0.93–1.15	4	1206	1.05	0.95–1.17	4	1198	0.99	0.88–1.11
	High	4	615	**1.25**	**1.02–1.52**	4	613	**1.14**	**1.00–1.29**	4	607	1.27	0.98–1.83

^a^ Model 1 = unadjusted. ^b^ Model 2 = adjusted for age, body mass index, smoking and leisure-time physical activity. ^c^ Model 3 = model 2 + additionally adjusted for educational level.

## Discussion

### Interpretation of the findings

The present IPD meta-analysis, based on data from a substantial dataset compiled from studies conducted in various European countries, provided insights into the association between OPA and both cardiovascular and all-cause mortality, stratified by different levels of CRF among male workers. Our study showed an association between high OPA levels and increased risks of cardiovascular mortality, particularly among those with low levels of CRF. This harmful association appeared to be lower among individuals with higher levels of CRF. However, a less clear pattern was found for all-cause mortality. Contrary to expectations, workers with high CRF exposed to high levels of OPA still had a 27% increased risk of all-cause mortality compared to workers with lower levels of OPA, although this increased risk did not reach statistical significance. This observation suggests that, even for male workers with high CRF, there may be an association with increased all-cause mortality due to high OPA, warranting further investigation.

Our findings thus suggest that high levels of CRF potentially mitigate the association between OPA and cardiovascular mortality, which is in line with the ‘fit for work’ paradigm. However, this paradigm was not supported for all-cause mortality. Further research involving larger populations of workers with high CRF will be necessary to establish a more conclusive understanding of the associations between OPA and all-cause mortality. Our meta-analysis contributes to the existing evidence by confirming previous findings on ischemic heart disease, cardiovascular and all-cause mortality (5, 6, 11, 21, 24, 36). Importantly, our study points once more to the importance of high levels of CRF in attenuating the adverse effects of high levels of OPA on cardiovascular mortality rates among male workers.

In the first IPD study (29), which utilized some of the same datasets as this analysis, we demonstrated that male workers with high OPA had an 12% increased risk of all-cause mortality compared to those with sedentary OPA. In that study, as well as in other research (13, 15, 29), potential mechanisms underlying the differential health outcomes of different physical activity domains were explored and discussed. With regard to the potential underlying mechanisms of the role that CRF can play in mitigating adverse effects of OPA, some physiological aspects can be mentioned. High levels of OPA have been associated with chronic exhaustion, elevated 24-hour blood pressure and heart rate (37), leading to accelerated progression of atherosclerosis (20) and an increased incidence of myocardial infarction (38) and thus an increased risk of (cardiovascular) mortality (6, 14, 25, 39). High CRF, which reflects the body’s efficiency in delivering oxygen to muscles during physical exertions, has been recognized as a protective factor for premature cardiovascular health problems (36, 40). Males with high CRF may have a greater ability to cope with the physical demands of OPA (41). Well-trained individuals typically exhibit a lower resting heart rate, reduced heart rate response to prolonged aerobic demands, and improved recovery between bouts of activity. These factors collectively enable consistent performance throughout work shifts characterized by elevated levels of physical activity, thereby reducing the risk of cardiovascular strain.

We found a possible attenuating effect of CRF on the association between OPA and cardiovascular mortality, but not for all-cause mortality. This difference could be explained by the direct impact of CRF on heart health and vascular function (42), which may be less clear for all-cause mortality. Individuals with better CRF tend to have healthier hearts, lower blood pressure, improved blood vessel function, favorable lipid profiles, and enhanced inflammatory response regulation (43–45). These factors all contribute to a reduced risk of cardiovascular diseases, which predominantly affects the heart and blood vessels.

The differences in effect, found in the present study of OPA on cardiovascular and all-cause mortality, can also be attributable to methodological factors. For the Cox regression analysis, the aggregated data revealed substantial differences in the weight assigned to the studies. For instance, in the comparison between low and high OPA, the Copenhagen Male Study contributed only 39% to the overall mortality results, whereas it contributed 71% to the pooled results for cardiovascular mortality. Given that the result of the Copenhagen Male Study was closest to a null effect, this study strongly influenced the results for cardiovascular mortality, whereas this effect was less pronounced for all-cause mortality. This highlights the susceptibility of outcomes to be influenced by individual studies, underscoring the importance of acquiring more studies with larger participant cohorts to achieve more robust results.

### Strengths and limitations

Our study was the first to investigate the association between OPA and both cardiovascular and all-cause mortality across different CRF groups by employing an IPD meta-analysis approach. In this approach, analytical methods were harmonized. The use of objectively measured CRF allows for a more precise assessment of individual aerobic fitness levels and provides a quantifiable assessment, eliminating potential biases associated with self-reported data. A further strength of this meta-analysis was that we could adjust for potentially important confounders such as LTPA, BMI, smoking and educational level.

Despite the advantages associated with IPD studies (46), this IPD also inevitably has some limitations. A first methodological concern is that the studies included in this meta-analysis were based only on self-reports of both OPA and many of the confounding factors. These factors were captured by means of crude categories, with notable variations in definitions across the included studies. This might lead to misclassification bias, in turn leading to an underestimation of the association between OPA and mortality (47). Additionally, all variables except for mortality were measured at baseline so changes over time, for example as participants transitioned out of active working ages, could not be taking into account. The long follow-up periods may have included post-retirement years, during which OPA health impact could diminish, potentially diluting observed associations. Second, while our goal was to collect data from a diverse sample of workers, it should be acknowledged that all studies only included participants from Western affluent nations. The generalizability of our findings to low- and middle-income countries is therefore limited. As the working conditions, living environment, and lifestyle in Western countries are generally more beneficial to health than in Southern and Eastern European countries, the observed harmful health associations with OPA can potentially be even stronger in the latter counties. Third, as our sample only consists of males, it is not possible to extrapolate our findings to females. Fourth, there might have been a healthy worker selection bias (48, 49). The healthiest workers are the ones who are most likely to be able to endure the highest levels of OPA and CRF, while individuals who suffer from (chronic) diseases or impaired health might have to shift to less strenuous job roles earlier in their career or even exit the workforce entirely. Fifth, potentially relevant confounders such as alcohol consumption, psychosocial work demands, and other work characteristics could unfortunately not be incorporated in the analyses. As we cannot rule out residual confounding, future studies should strive for better ways to take care of confounding in the association of physical activity and health-related outcomes. Sixth, although CRF was objectively measured in each of the original studies, the methods used to measure CRF varied across studies. Four studies expressed CRF as VO_2_max, while one study used watts/kg. This variability in measurement methods, as outlined in table 1 and supplementary table S3, means that the classification of CRF into tertiles is study-specific and dependent on the characteristics of each study population. The internal validity of the CRF tests differs between studies, which may affect the results. Additionally, the approach of using tertiles leads to a classification that is not based on absolute clinical numbers, potentially resulting in misclassification. Approximately 25% of participants did not participate in CRF measurements and some did not complete the questionnaires, resulting in missing information for certain confounder variables (<2%). This missing data may have introduced selection bias, as the final dataset may not fully represent the original population. This, in turn, could affect the generalizability of our findings.

### Practical implications

Based on the findings of the present study, enhancing the CRF levels of workers engaged in high OPA appears important for reducing the potential risk of cardiovascular mortality. This recommendation is consistent with previous studies advocating for increased CRF levels to mitigate negative health outcomes associated with OPA (10, 50). Integrating fitness activities during work or leisure time can facilitate this goal; however, implementing high-intensity physical activities during leisure time can be challenging due to the physical strain of their jobs and limited recovery time.

Using the workplace as a platform for improving workers’ health has been suggested as an effective strategy (50–53), particularly for engaging hard-to-motivate groups such as workers with low CRF levels. Workplace health interventions, such as on-site aerobic training sessions, have proven effective in enhancing CRF across various professions (54, 55). It is crucial to ensure that work environments are conducive for all employees, regardless of their current fitness levels. Emphasizing improvements in working conditions and practices could make physical demands more manageable for everyone, promoting inclusivity and addressing health disparities in occupational settings.

For workers lacking cultural capital or resources, personalized and tailored advice becomes essential (56). Achieving a balanced equilibrium between OPA and individual capacity, as indicated by CRF, may involve optimizing work tasks and schedules to allow for adequate energy replenishment and reduce physical strain. By focusing on improving work environments and practices, organizations can create a more supportive and health-promoting atmosphere for all employees.

### Future research

To advance our understanding of the interplay between OPA, CRF, and health-related outcomes, future studies would benefit from implementing methodological improvements aimed at reducing exposure misclassification bias and enhancing the precision of findings. This could be achieved by combining self-reported and devise-based measures (eg, accelerometers or inclinometers) to quantify both the biomechanical and the aerobic aspects of the type, intensity and duration of OPA with logs or self-reported diaries regarding time use. Additionally, further investigations are needed to explore the underlying mechanisms that might be responsible for the observed cardiovascular effects of high OPA and potentially preventive role of CRF. To optimize cardiovascular health outcomes and reduce physical strain, interventions could focus on reducing relative aerobic workloads, incorporating a combination of aerobic exercise and ergonomic adjustments, such as the integration of robotics technology, while concurrently promoting improved CRF.

### Concluding remarks

Our IPD meta-analysis showed that males with low levels of CRF and high levels of OPA have a significantly elevated risk of cardiovascular and all-cause mortality. Among those with high CRF, the risk of cardiovascular mortality associated with high OPA appears to be mitigated; however, this was not observed for all-cause mortality. This finding underscores the potential role of CRF in mitigating cardiovascular mortality risks and supports the ‘fit for work’ principle in that context.

## Supplementary material

Supplementary material
