# Supplementary material for: Exploring the ‘fit for work’ principle: The association between occupational physical activity, cardio-respiratory fitness, and mortality – a meta-analysis of male worker data
Source: Scand J Work Environ Health. 2025 Apr 27;51(3):159–69. doi: 10.5271/sjweh.4218 (PMC12047211; doi:10.5271/sjweh.4218)

Exploring the 'fit for work' principle: The association between occupational physical activity, cardio-respiratory fitness, and mortality – a meta-analysis of male worker data [1]

by Margo Ketels, PhD,[2] Bart Cillekens, PhD, Els Clays, PhD, Maaike A Huysmans, PhD, Dirk De Bacquer, PhD, Andreas Holtermann, PhD, Richard P Troiano, PhD, Paul Jarle Mork, PhD, Steinar Krokstad, PhD, Henry Völzke, PhD, Marcus Dörr, PhD, Martin Bahls, PhD, Till Ittermann, PhD, Johan Clausen, PhD, Magnus T Jensen, PhD, Jussi Kauhanen, PhD, Ari Voutilainen, PhD, Miriam Wanner, PhD, Matthias Bopp, PhD, Willem van Mechelen, PhD, Allard J van der Beek, PhD, Pieter Coenen, PhD

1. Supplementary material
2. Correspondence to: Dr Pieter Coenen, Department of Public and Occupational Health, Amsterdam UMC, van der Boechorststraat 7, 1081 BT Amsterdam, the Netherlands. [E-mail: p.coenen@amsterdamumc.nl]

Supplementary Table S1. Overview of included studies, their main characteristics and risk of bias.

			Sample information										Risk of bias			
Reference	Study name	Country	N	Age at baseline	Sample	Base line	Mean follow -up period	ICD codes for cardiovascular mortality	Cumulative incidence, N (%)		Incidence rates, per 10 000 person years		1	2	3	4
									All-cause mortality	Cardiovascular mortality	All-cause mortality	Cardiovascular mortality				
Clays et al., 2014	BELFIT study	Belgium	1498	46.3	Industry	1976	16.7	ICD 9: 401-444	153 (10.2)	39 (2.6)	60.6	15.4	Mod	Mod	Mod	Low
Krause et al., 2017	KIHD study	Finland	1737	52.0	Population	1984	24.7	ICD 10: I00-I99	863 (49.7)	381 (21.9)	200.7	88.6	Low	Low	Low	Low
Bahls et al., 2018	SHIP-START1 study	Germany	437	44.8	Population	2002	8.2	ICD 10: I10-I79	11 (2.5)	2 (0.5)	30.7	5.6	Mod	Mod	Low	Low
Wanner et al., 2014	NRP 1A study	Switzerland	1040	40.5	Population	1977	32.5	ICD 10: I00-I99	423 (40.7)	147 (14.1)	125.3	43.6	Mod	Low	Mod	Low
Holtermann et al., 2009	Copen hagen male study	Denmark	5210	48.8	Workers	1970	28.4	ICD 8: 400-448<br>ICD 10: I00-I99	4833 (92.1)	2243 (42.6)	326.1	151.3	Mod	Low	Mod	Low
			9922	46.5			22.1		7,763 (50%)	3,267 (21%)						

Age in years (mean (SD); FU = Follow-up period (years); ICD = International classification of Disease

1=Study participation; 2=Study attrition; 3=Predictive variable assessment; 4=Outcome ascertainment. Risk of bias was assessed according to established criteria (Hayden et al., 2013).

Supplementary Table S2. Risk of bias of the studies included in the individual data meta-analysis (adapted from Coenen et al. submitted)

Reference	Study name	Study participation		Study attrition		Predicting variable measurement		Outcome	
		**Assessment**	**Explanation**	**Assessment**	**Explanation**	**Assessment**	**Explanation**	**Assessment**	**Explanation**
Clays et al., 2014	BELFIT study	Moderate risk	Unclear how participating companies were selected. Also, a substantial part of the selected participant (25%) did not participate	Moderate risk	Of 2,363 eligible workers, 1456 were analyzed. Loss to follow-up is unclear	Moderate risk	Accuracy of the occupational physical activity questionnaire is unclear. The Minnesota Leisure-time Physical Activity Questionnaire has shown reasonable accuracy (Taylor et al., 1978)	Low risk	Ascertainment through registry seems adequate
Krause et al., 2017	KIHD study	Low risk	There was an 83% participation rate and an adequate description of the sample (Supplementary Table S1)	Low risk	There was no loss of follow-up. All working participants were included and National Finnish death registries have complete data for all Finnish residents	Low risk	Occupational physical activity questionnaire showed good reliability (test-retest correlation 0.69[63]). The Minnesota Leisure-time Physical Activity Questionnaire has shown reasonable accuracy (Taylor et al., 1978)	Low risk	Ascertainment through registry seems adequate

Bahls et al., 2018	SHIP-START1 study	Moderate risk	A random sample of the population was drawn, with 68% response rate. Adequate description of the sample is provided (Supplementary Table S1)	Moderate risk	Unclear how many participants were lost to follow-up	Low risk	Baecke questionnaire was used which has shown reasonable accuracy (Philippaerts et al., 1999)	Low risk	Ascertainment through registry seems adequate
Wanner et al., 2014	NRP 1A study	Moderate risk	Sampling procedure is unclear (with a combination of a random and convenience sample used).	Low risk	93% of participants were followed up (while another 500 participants were excluded due to missing data)	Moderate risk	Accuracy (i.e., validity) of questions is unclear	Low risk	Ascertainment through national registry seems adequate
Holtermann et al., 2009	Copenhagen male study	Moderate risk	High response rate (87%) and good description of the sample. Sample of workers from various companies.	Low risk	Only nine men had missing answers, and 14 had emigrated during the follow-up	Moderate risk	The questionnaire was earlier used. However, its origin and accuracy is unclear.	Low risk	Ascertainment through registry seems adequate

Supplementary Table S3. Measurement of Cardio-respiratory fitness.

**Author**	Study name	CRF measurement	Outcome expressed as
Clays et al., 2014	BELFIT study	Submaximal graded exercise test on a bicycle ergometer.<br><br>The initial workload was fixed at 75 Watts, with 25-Watt increment every 2.5 minutes. The target heart rate at which the test was terminated (?) was 150 beats/minutes corresponding to 80% of the predicted maximal heart rate in the study population.<br><br>Physical working capacity was defined as the work load, expressed in watts, at a heart rate of 150 beats/minute and was calculated by interpolation. The physical working capacity value was standardized for body weight and was used as the criterion for cardiorespiratory fitness	Watts/kg
Krause et al., 2017	KIHD study	Cardiorespiratory fitness was assessed by a symptom-limited maximal (?) exercise test on an electrically braked 400 L-cycle ergometer (Medical Fitness, Mearn, the Netherlands)<br><br>The exercise tests were performed between 8:00 a.m. and 10:00 a.m. by using a standardized testing protocol, with a linear increase in the workload by 20 $W.min^{-1}$. Oxygen consumption was measured by using the breath-by-breath method of respiratory gas exchange (Medical Graphics, St. Paul, Minnesota). The VO_2 max was defined as the highest value or the plateau in oxygen uptake and was expressed per kg. body weight per minute.	VO_2max ($ml.kg^{-1}.min^{-1}$)
Bahls et al., 2018	SHIP-START1 study	A modified Jones protocol using a calibrated electromagnetically braked cycle ergometer (Ergoselect 100, Ergoline, Germany).<br><br>After 3 minutes of unloaded cycling plus the ergometer related permanent load, work load increased step-wise at a rate of 16 $Watts.min^{-1}$. Gas exchange and ventilatory variables were analyzed breath by breath averaged over 10-second intervals using a computer-based system.<br><br>VO_2peak was defined as the highest 10-second average of VO_2 in the last minute of maximal (?) exercise. The anaerobic threshold (VO2@AT) was based on non-invasive determination by gas exchange analysis by assessing the relation of VO_2 to VCO_2 (V-slope method).<br><br>W_{max} was operationalized as the highest reached power on the bicycle ergometer during exercise kept for at least 20 s at VO2peak.	VO_2max ($ml.kg^{-1}.min^{-1}$)
Wanner et al., 2014	NRP 1A study	In the NRP 1A study, Cardiorespiratory Fitness (CRF) was assessed using an ergometric score that accounted for age and sex. The ergometric test, typically lasting 6 minutes, measured heart rate at various points, including the average heart rate during the last two minutes, expressed in beats per minute. Power output, expressed in watts by the braking weight, was also recorded. The ergometric score was calculated from these variables—heart rate, average heart rate, power output, age, and sex—and rounded up to the nearest whole number if the decimal was 0.5 or higher.	VO_2max ($ml.kg^{-1}.min^{-1}$)
Holtermann et al., 2009	Copenhagen male study	Aerobic fitness was determined using information on heart rate and work load from a submaximal bicycle ergometer test and the Åstrand nomogram.<br><br>The load chosen in each case was determined by the body weight	VO_2max ($ml.kg^{-1}.min^{-1}$)

		and age of the person or heart rate during the first minute of the test; VO_2max was estimated using Åstrand's nomogram.	

Supplementary Figure 1. OPA and risk of cardiovascular mortality (first graph) and all-cause mortality (second graph) according to cardiorespiratory fitness level: lowest tertile, medium tertile and highest tertile. Cox regression models were adjusted for age, smoking, BMI, LTPA and education.

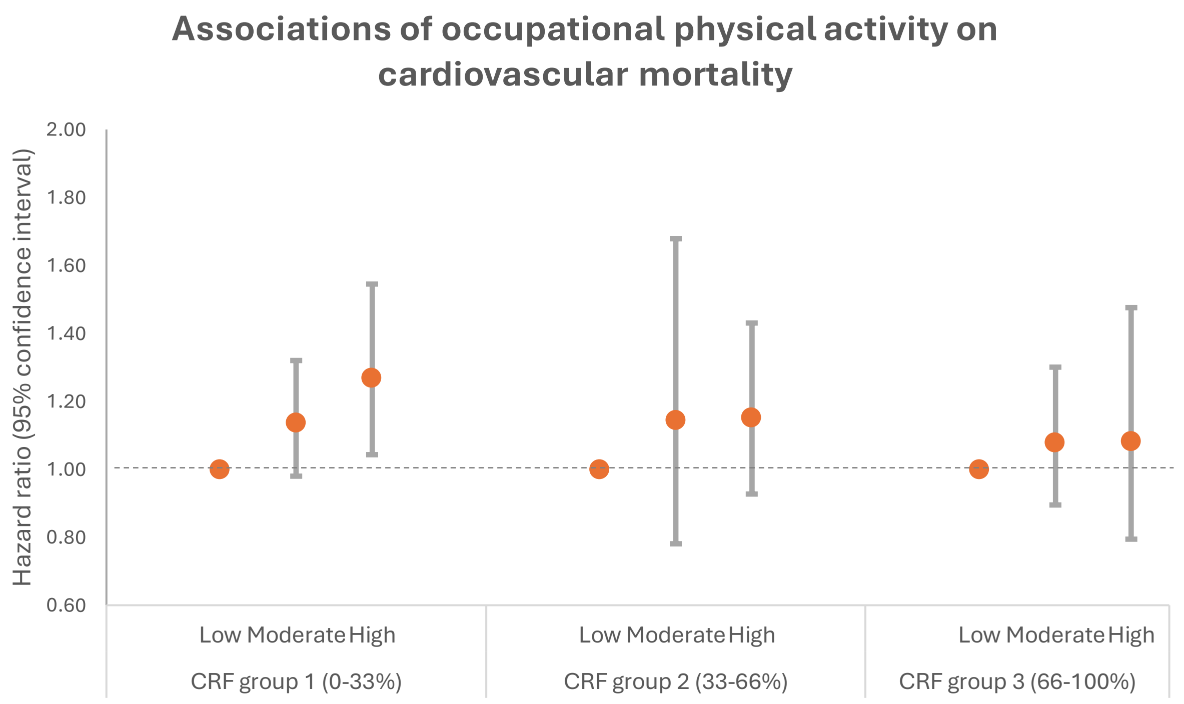

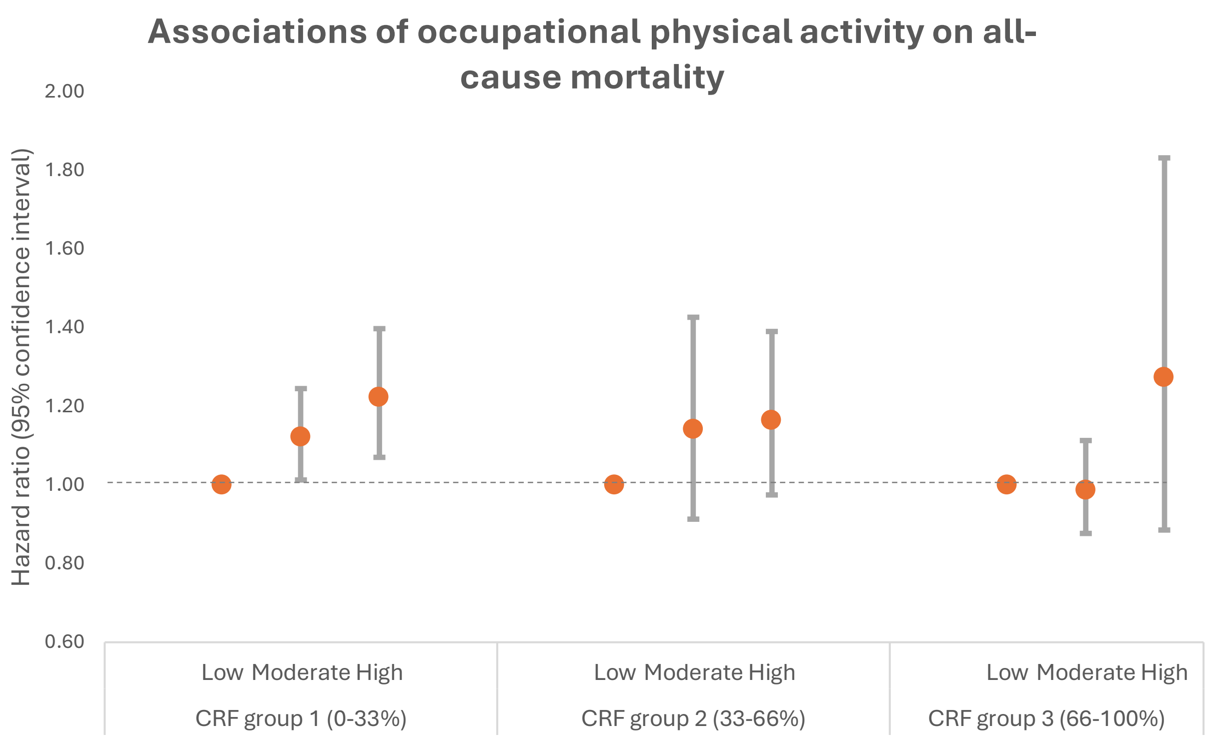

Supplementary Figure 2. Forest plots depicting the individual study and pooled association of occupational physical activity with cardiovascular mortality across the CRF groups. We report individual study effect sizes, their weight in the pooled effect size, and the pooled effect size depicted in the diamond.

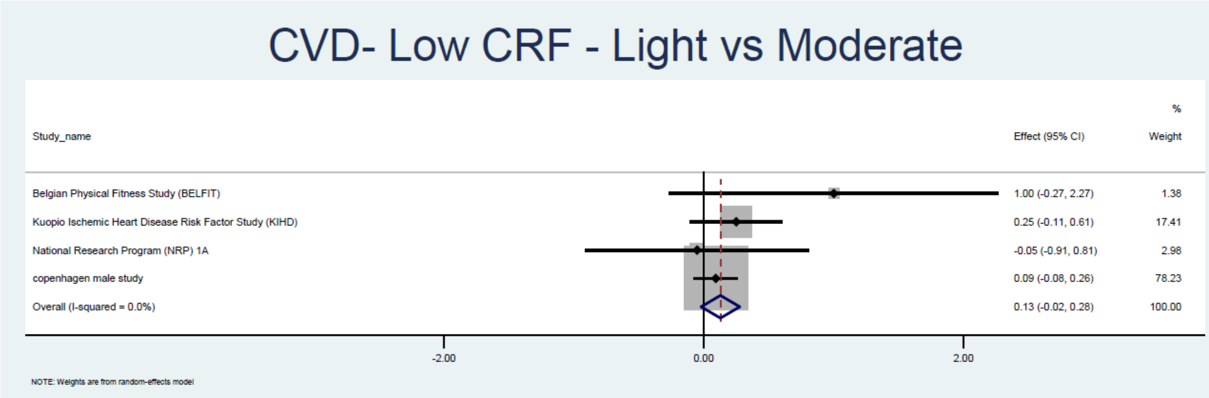

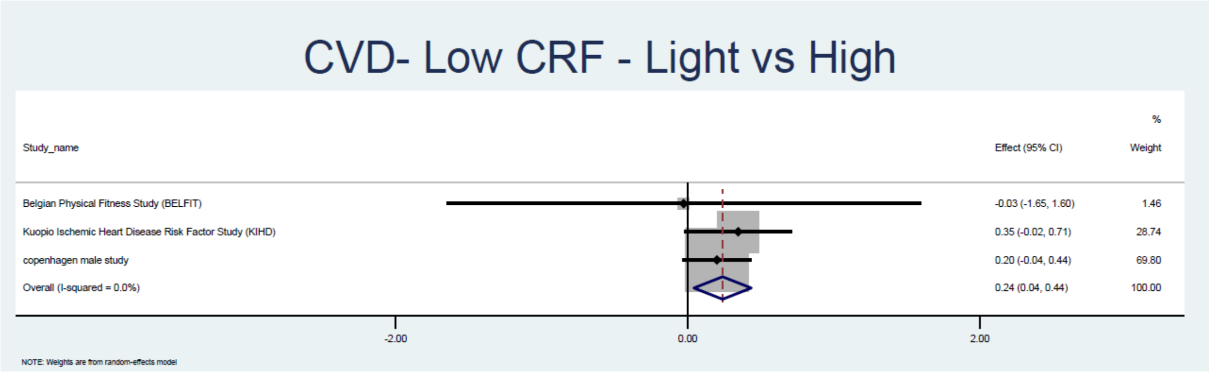

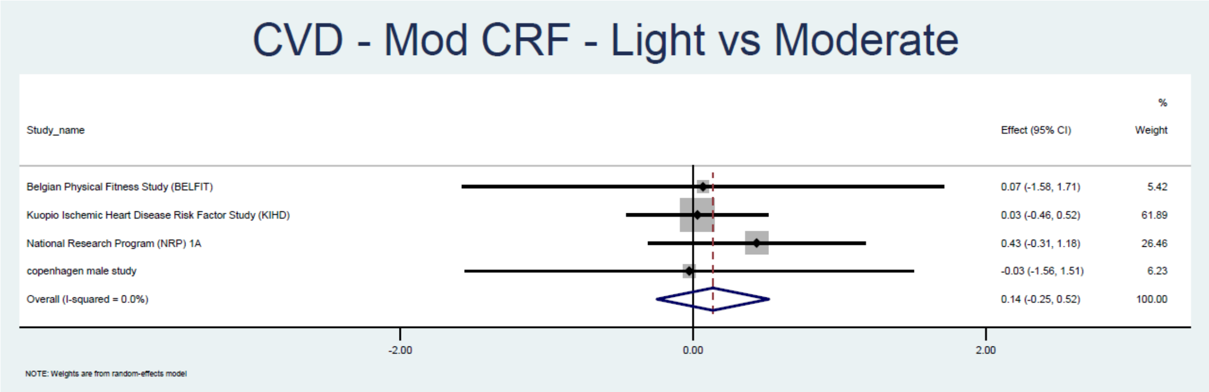

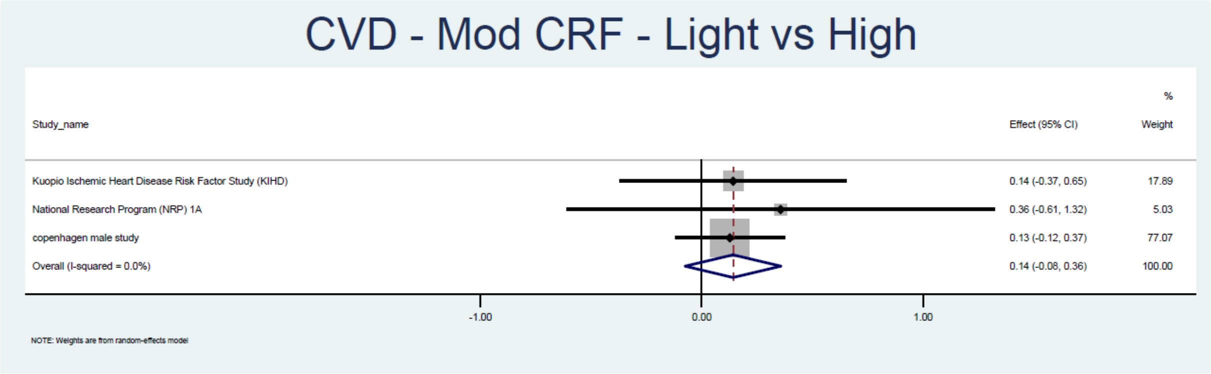
CVD - Mod CRF - Light vs High
%
Study_name
Effect (95% CI)
Weight
Kuopio Ischemic Heart Disease Risk Factor Study (KIHD)
0.14 (-0.37, 0.65)
17.89
National Research Program (NRP) 1A
0.36 (-0.61, 1.32)
5.03
copenhagen male study
0.13 (-0.12, 0.37)
77.07
Overall (I-squared = 0.0%)
0.14 (-0.08, 0.36)
100.00
-1.00
0.00
1.00
NOTE: Weights are from random-effects model

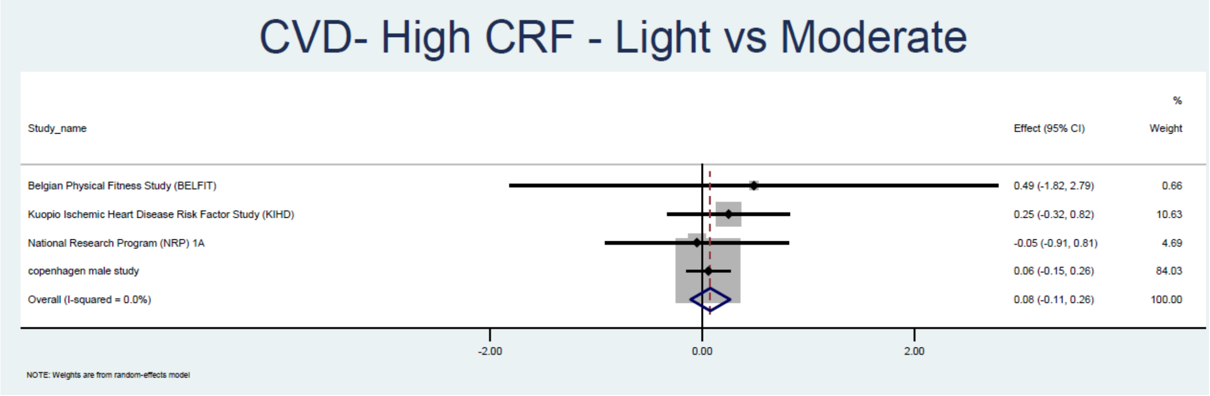
CVD- High CRF - Light vs Moderate
%
Study_name
Effect (95% CI)
Weight
Belgian Physical Fitness Study (BELFIT)
0.49 (-1.82, 2.79)
0.66
Kuopio Ischemic Heart Disease Risk Factor Study (KIHD)
0.25 (-0.32, 0.82)
10.63
National Research Program (NRP) 1A
-0.05 (-0.91, 0.81)
4.69
copenhagen male study
0.06 (-0.15, 0.26)
84.03
Overall (I-squared = 0.0%)
0.08 (-0.11, 0.26)
100.00
-2.00
0.00
2.00
NOTE: Weights are from random-effects model

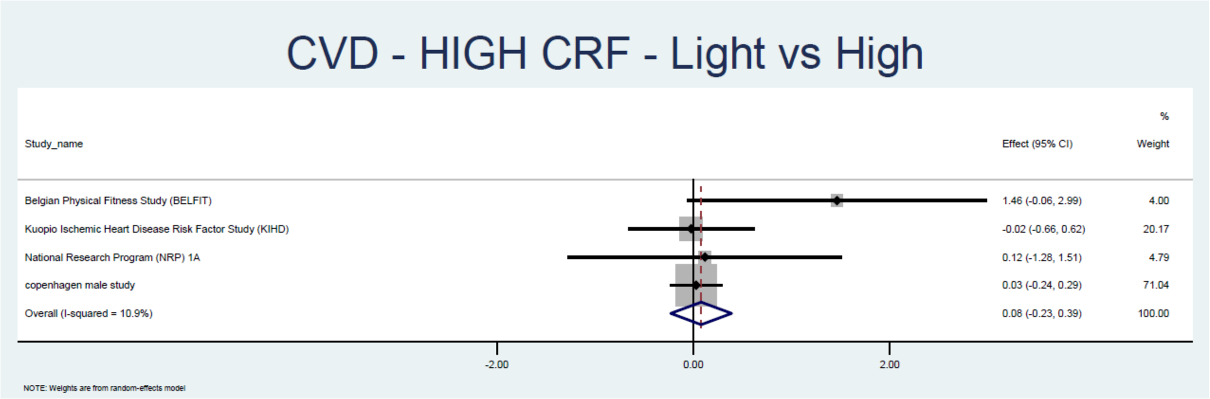
CVD - HIGH CRF - Light vs High
%
Study_name
Effect (95% CI)
Weight
Belgian Physical Fitness Study (BELFIT)
1.46 (-0.06, 2.99)
4.00
Kuopio Ischemic Heart Disease Risk Factor Study (KIHD)
-0.02 (-0.66, 0.62)
20.17
National Research Program (NRP) 1A
0.12 (-1.28, 1.51)
4.79
copenhagen male study
0.03 (-0.24, 0.29)
71.04
Overall (I-squared = 10.9%)
0.08 (-0.23, 0.39)
100.00
-2.00
0.00
2.00
NOTE: Weights are from random-effects model

Supplementary Figure 3: Forest plots depicting the individual study and pooled association of occupational physical activity with all-cause mortality across the CRF groups. We report individual study effect sizes, their weight in the pooled effect size, and the pooled effect size depicted in the diamond.

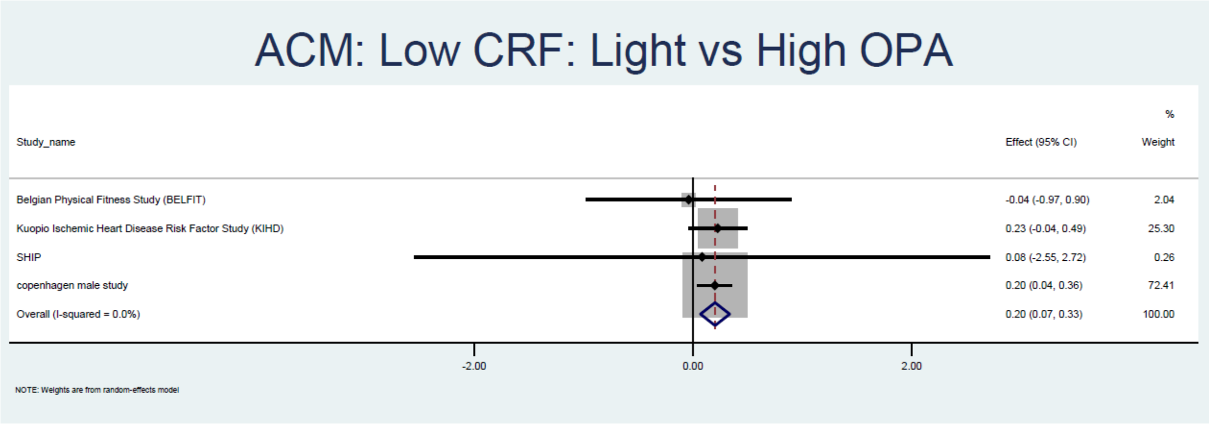

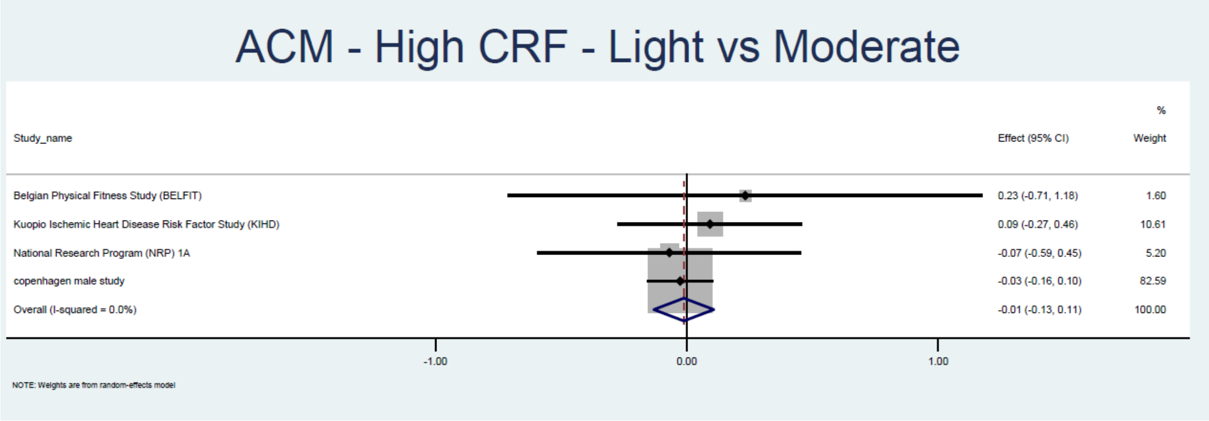

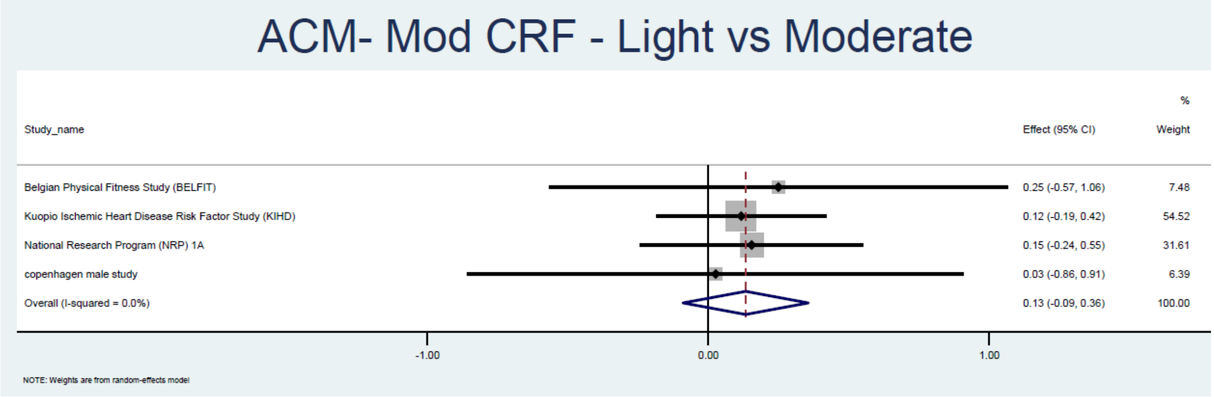

ACM- MOD CRF - Light vs High

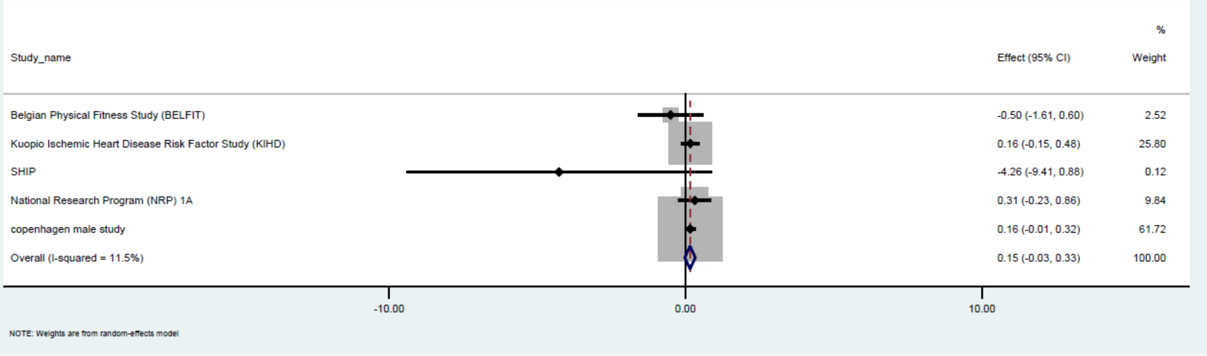

ACM - High CRF - Light vs Moderate

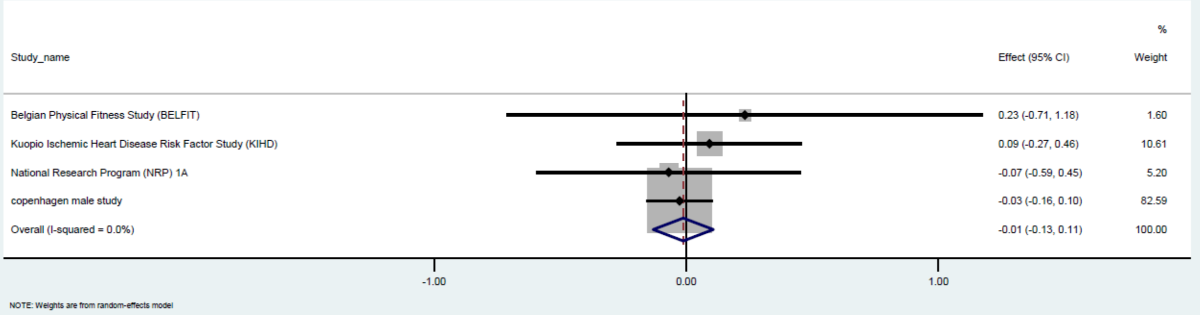

ACM - High CRF- Light vs High

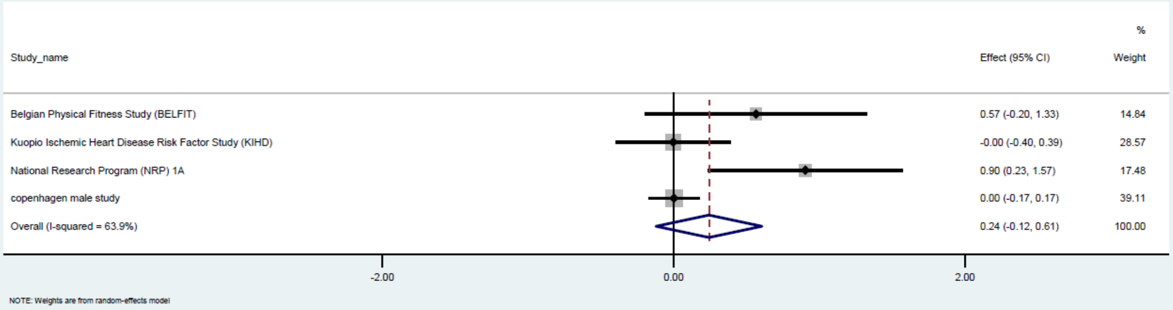

Supplement: Supplementary material [file SJWEH-51-159-S001.pdf]
